# Diagnosis experiences from 50 hepatitis B patients in Chongqing, China: a qualitative study

**DOI:** 10.1186/s12889-021-11929-9

**Published:** 2021-12-01

**Authors:** Xiangxi Zhou, Fan Zhang, Yongping Ao, Chunli Lu, Tingting Li, Xianglong Xu, Huan Zeng

**Affiliations:** 1 School of Public Health and Management, Chongqing Medical University, No. 1 Yixueyuan Road, Yuzhong District, Chongqing, 400016 China; 2 Research Center for Medicine and Social Governance in Health, Chongqing Medical University, Chongqing, China; 3 The Innovation Center for Social Risk Governance in Health, Chongqing Medical University, Chongqing, China; 4 Centers for Disease Control and Prevention of Chongqing, Chongqing, China; 5 Central Clinical School, Faculty of Medicine, Nursing and Health Sciences, Monash University, Melbourne, Australia; 6 Melbourne Sexual Health Centre, Alfred Health, Melbourne, Australia; 7 China Australia Joint Research Center for Infectious Diseases, School of Public Health, Xi’an Jiaotong University Health Science Centre, Xi’an, Shaanxi People’s Republic of China

**Keywords:** Hepatitis B, Diagnosis experience, Liver funtion test, Qualitative study

## Abstract

**Background:**

The aim of this study was to provide recommendations for reducing the impact of hepatitis B infection on patients with chronic hepatitis B by describing their experiences during the diagnosis process.

**Methods:**

We conducted face-to-face interviews with 50 hepatitis B patients recruited by convenient sampling from an infectious diseases department of a teaching hospital in Chongqing, China from July to August 2019. Thematic analysis framework included interviewees’ social demographic characteristics, diagnosis approach, signs and symptoms before diagnosis, feelings after diagnosis, and doctor’s instructions.

**Results:**

Most patients first detected hepatitis B through various types of physical examinations when the patients were asymptomatic or had only mild symptom**s. Most** patients were shocked, scared, or overwhelmed when they were diagnosed with hepatitis B. They were able to remember the doctor’s instructions about maintaining a healthy lifestyle, but not impressed by the doctor’s advice about regular follow-up liver function tests. The lack of regular follow-up has caused irreversible damage to some patients.

**Conclusions:**

Most patients are passively diagnosed with hepatitis B due to their lack of awareness on active hepatitis B prevention. Patients need professional mental health care to overcome the negative emotions that following the diagnosis. Physicians’ instruction should emphasize the importance of regular follow-up liver function tests in addition to a healthy lifestyle.

## Background

Known as a global health problem with a significant disease burden, chronic hepatitis B has a long course and can lead to many adverse outcomes, including liver cirrhosis and liver cancer [1]. An estimated 350 million persons are chronically infected with hepatitis B virus (HBV) worldwide, resulting in 600,000 deaths annually from the complicating diseases. It is reported that chronic hepatitis B infection is related to one third of all liver cancer cases in the world [2]. Presently in China, hepatitis B is a severe public health issue [3], as the large number of hepatitis B cases has brought a heavy economic burden on the patients and their families. In 2016, the WHO proposed to eliminate hepatitis B by 2030, and the Chinese government has also declared a strong determination to achieve this goal [4–6]. Recent studies have reported that the incidence of hepatitis B has greatly decreased during the last decade [7, 8], while approximately 120 million people remain hepatitis B virus (HBV) tests positive [9–12]. As a developing country with a large number of patients infected with HBV, China would make great contribution to the goal of eliminating hepatitis B worldwide by 2030 [13]. Given the high prevalence of hepatitis B in China, both the diagnosis and treatment rate are relatively low [14, 15]. According to the data from National Disease Supervision Information Management System of China, the reported mean incidence of hepatitis B was 84.3 per 100,000 people between 2005 and 2010 [16]. It is reported that only 58.2% of hepatitis B patients had been received antiviral medications in 2011 [17]. Moreover, studies have found that hepatitis B is asymptomatic at its early stage [18]. Therefore, early detection, professional treatment, and regular follow-up are especially important to achieve positive prognoses for the patients.

Understanding the diagnostic process of hepatitis B is essential to detect the specific barriers to hepatitis prevention and control, thus to formulate effective measures. It is known that HBV can be transmitted from mother to child, blood (including minor skin and mucosal wounds) and sexual contact [19]. A patient can be diagnosed with hepatitis B based on blood transfusion history, contact history with HBV-infected patients or family members. Most importantly, laboratory examinations on hepatitis B surface antigen (HBsAg), hepatitis B surface antibody (HBsAb) and hepatitis B core antibody (HBcAb) are needed to confirm the diagnosis. In order to achieve the goal of eliminating hepatitis B by 2030, China should make efforts to improve the diagnosis and treatment rates. Early detection, early diagnosis and early treatment is crucial to curb hepatitis B.

Many researchers have investigated the prevalence of hepatitis B in China. Some studies have described a decreasing trend in the prevalence of hepatitis B among young people and an increasing trend among the elderly [20–22]. After searching major literature databases such as CNKI, VIP, CBM, and PubMed, we could not found articles describing the diagnosis experiences of hepatitis B patients. In this study, we reported findings from a qualitative study with hepatitis B patients to describe their experiences on hepatitis B test, symptoms and signs before diagnosis, feeling when being diagnosed and the doctor’s advice. Mapping the case finding pathways and the specific problems that exist in the diagnosis process could contributed to formulating evidence-based strategies.

## Materials and methods

### Setting and population

This study was conducted in Chongqing, which is located in the southwest of China and is one of the four municipality cities nationwide. We selected the infectious disease department of a teaching hospital affiliated to a medical university to recruit participants. Patients diagnosed with chronic hepatitis B in this department were chosen as participants. An in-depth qualitative investigation was conducted from July 2019 to August 2019.

### Data collection

This research included a total of 50 participants through convenience sampling. All interviews were conducted face-to-face by three interviewers between July 2019 and August 2019 (one acted as the primary interviewer, the others were responsible for observation and taking detailed notes). First, the interview team defined the questions in advance. Six aspects were explored for this study, including social-demographic details, ways of HBV detection, test results, signs and symptoms, feelings at the time of diagnosis, and physician orders. In addition, social-demographic information such as gender, age, education, occupation, marital status, place of residence, and carriers in the household was requested. The questions and responses of the participants were then recorded in qualitative research group interviews and the transcripts were then analyzed.

Prior to each interview, participants were informed of the study purpose, their rights to withdraw at any time, and the potential risks and benefits of participation in the study. Each interview lasted approximately 30–60 min. Patients’ names were removed to ensure confidentiality. The semi-structured interview was conducted according to the guidelines and regulations from the ethics committee of Chongqing Medical University.

### Data analysis

The recorded interviews were firstly transcribed into Mandarin, then two researchers read through the transcripts carefully and listed the recurring themes. Then a thematic framework was established based on the themes and the topic guide used in the interviews. Later, all the transcirptions were imported into a document where they were coded line by line according to the thematic framework. The coded segments under each theme were then summarised and synthesised into an organised chart. Finally, the illustrative quotes selected for the results were first translated into English and then back-translated to Chinese by two researchers independently to assure the quality of translation and consistency.

## Results

### Social-demographic characteristics of the participants

A total of 50 participants diagnosed with hepatitis B were enrolled in this study. Table 1 shows the social-demographic characteristics of the participants. The majority of them were above 30 years old. Most participants received junior high school, senior high school, and undergraduate course (the percentages were 24, 16, and 18%, respectively). The most common occupation was workers (12%), following by the self-employed (10%), farmer (8%), office staff (8%), and civil servant (8%). Most of the participants lived in Chongqing (70%), and approximately a half (26/50) had family history of hepatitis B.

**Table 1:** Social-demographic characteristics of the participants

Demographics	N(%)	Demographics	N(%)
**Age (years)**		**Gender**	
≤ 19	1(2)	Male	30(60)
20–29	8(16)	Female	20(40)
30–39	15(30)	**Occupation**	
40–49	11(22)	Barber	1(2)
50–59	12(24)	Civil servant	4(8)
≥ 60	1(2)	Company manager	7(14)
No record	2(4)	Do odd jobs	1(2)
		Driver	1(2)
**Education**		Farmer	5(10)
Primary school	2(4)	No job	1(2)
Junior high school	20(40)	Office staff	2(4)
High school	13(26)	Retiree	2(4)
Undergraduate	11(22)	Salesman	2(4)
Master	4(8)	Self-employed laborer	8(16)
		Student	2(4)
**Location**		Teacher	3(6)
Chongqing	35(70)	Technician	7(14)
Sichuan	5(10)	Worker in company/factory	4(8)
Guizhou	2(4)	**Does the family have a carrier or case**	
Hunan	4(8)	Yes	26(52)
Other places	1(2)	No	21(42)
No record	3(6)	No record	3(6)

### How did you find out you had been infected with HBV?

As shown in Table 2, the participants had been found themselves hepatitis B virus (HBV) infection from six ways. Nearly a half(22/50) was detected by specific physical examinations, including personal health examination, pregnancy check-ups, physical examination for college admission or employment, routine physical examination for schoool students or workers in factory/company. A noticeable proportion (15/50) was identified after their family members/neighbors/friends being diagnosed hepatitis B. Specially, only a small percentage(6/50) went to test by themselves since the emergence of mild disease symptoms.

**Table 2:** How do the participants being found hepatitis B infection

	N
**How to being found hepatitis B virus infection**(*n* = 50)	
Specific physical examinations in different situations	22
Go to test after family members diagnosed hepatitis B	8
Go to test after friends or neighbors being found hepatitis B	7
Go to test because of mild disease symptoms	6
Being diagnosed when sick in hospital because of other diseases	5
Test before blood donation/application for medical certificate of fitness	2
**Classification of specific physical examinations**(*n* = 22)	
Personal health examination	4
Pregnancy check-ups/Antenatal examination	4
Routine physical examination for workers in factory/company	5
Physical examination after being employed	2
University entrance physical examination	5
Routine physical examination for schoool students	2

#### Specific physical examination

In 2004, companies focusing on enterprise health management began to appear on the market in China. In 2006, companies for personal health management emerged. In 2010, China issued corresponding laws and regulations to advocate regular physical examination. In 2011, a large number of health management companies emerged in the market. According to a respondent who has had a personal physical examination, *“I went to check the hepatitis B antibody in May 2018. I had never done physical examination before. I found that I was infected with hepatitis B virus in my first physical examination. “ (YA 15,54 years old,female patient).* A new patient reported that *“half a year ago, I was found to have hepatitis B in the hospital of Dazu County. At that time, I felt a little tired, and I didn’t get any other symptoms. “ (YA 28,32 years old,male patient)* Other patients described as *“in 2012, I was firstly found hepatitis B because of physical examination, now I have been sick for nearly eight years, but physical examination showed no abnormality, no symptoms.” (YA 42,50 years old,male patient)* Another patient mentioned that *‘It was discovered in the physical examination in 1994 or 1993. It has been more than twenty years. I have no symptoms and I do not care about the infection.” (YA 20,48 years old, female patient).*

In 2001, *The Maternal and Child Health Care Law* began to be implemented in China, which requires medical and health institutions to provide free and basic prenatal care for all pregnant women in the country. In 2010, free hepatitis B screening was added to prenatal examination, but before that, it was not required. One interviewee who was detected hepatitis B through prenatal examination presented, *“I was found a carrier of hepatitis B virus when I was pregnant more than ten years ago. At present, my test indicated that hepatitis B surface antigen is positive and liver function is abnormal.” (YA 27,57 years old, female patient).*

In China, before 2010, HBV test is required in routine physical examination for workers in factory or company and physical examination after being employed. One patient expressed that *“when I worked in Guangzhou, I participated the routine physical examination organized by our factory, the HBV test result was normal. But since last year, my transaminase was found elevated.” (YA 11,53 years old, female patient)* Hepatitis B screening was also required for the entrance physical examination of universities in China between 2005 and 2010. As one patient mentioned *“I was diagnosed with HBV-positive in the university entrance physical examination.” (YA 09,34 years old, female patient)* The school routine physical examination started in 2007 in China. One patient said “*in the first day of my junior high school, we had a physical examination and then knew that the level of my transaminase was relatively high*. *“(YA 25,21 years old, male patient)* Another patient described *“when I was a child in school, I was found as a hepatitis B carrier. My liver function was normal, I needed not to be treated.” (YA 22,34 years old, female patient).*

#### Go for test after family members being diagnosed hepatitis B

There were eight patients decided to take a HBV test and found themselves HBV infection after their family members were diagnosed with hepatitis B. Among them, three took the advice on test from a doctor and the remaining five took the advice from their families. One patient said *“After graduation from high school, considering my father was diagnosed hepatitis B, then I went to the hospital to do a test and found that I was also infected with HBV.” (YA 35,46 years old, male patient)* Another patient said*“When I was 18 years old ,my mother got hepatitis B, and the doctor suggested that I should also take a HBV test. The doctor did not recommend any medication based on my test results. “ (YA 16,29 years old, male patient).*

#### Go for test after friends or neighbors being found hepatitis B

Seven of the participants went to the hospital to do HBV test after they heard that their neighbors or friends were HBV positive and finally confirmed that they also had HBV infection. A male patient described*“In 2009, I went to the county hospital for physical examination. Because of the high prevalence of hepatitis B in my village at that time and the fact that many people died from liver cancer, I decided to do a physical examination and finally found that I was sick.”(YA 03,40 years old, male patient).*

#### Go to test because of mild disease symptoms

Six patients were diagnosed with the HBV infection after they noticed some mild symptoms including fatigue, nausea, vomiting, and weakness. Patients recalled:*“In 2004, I felt uncomfortable, and the main manifestation was soft feet. I went to the hospital to do a physical examination and I was told that my liver function was abnormal. “(YA 32,male patient) “I had symptoms and felt uncomfortable. I thought it was problem of my stomach. Then I was diagnosed with hepatitis B.” (YA 41,55 years old, female patient).*

#### Being diagnosed when sick in hospital because of other diseases

A small number of patients were diagnosed with hepatitis B while they were examined for other diseases. One patient said *“my hepatitis B was first identified during a blood test in a hospital because of a cold, and was eventually diagnosed in 2017 when I was in hospital due to cerebral hemorrhage” (YA 08,51 years old, male patient).*

#### Test before blood donation/application for medical certificate of fitness

Only two patients found out that they had hepatitis B when they donated blood or applied for a medical certificate of fitness. They all described that they were not aware of it when they were infected with HBV. One female patient said*“While donating blood on the local street, I was taken a small amount of blood for pre-examination. Then I was told that I had HBV infection.”(YA 01,42 years old, female patient)* Another patient remembered that *“while applying a medical certificate of fitness two years ago, I found my liver function abnormal by a blood test. Then I was diagnosed with HBV infection at the First Affiliated Hospital of Chongqing Medical University.” (YA 38,30 years old, male patient).*

### Patient’s signs and symptoms before diagnosis

Many participants reported that they had no obvious symptoms related to hepatitis B before diagnosis. One participant said *“At that time, I felt no discomfort, so I didn’t feel bad.”* The early symptoms they referred mainly include nausea, decreased sleep quality, fatigue and indigestion. Before diagnosis, patients had no or few symptoms similar to those of other diseases, so hepatitis B can be easily overlooked. Years later, if for many reasons, the patients failed to take liver function tests to detect the possible pathologic changes, some patients may suffer permanent liver damage. Patients recollected*“At that time, there was no physical discomfort, so I didn’t feel anything. Later, hepatitis B was detected.”(YA 01,42 years old, female patient) “Two years ago, I had a bad stomach and slept poorly. Since I lived in a rural area at the time, I didn’t go for check-ups nor pay any attention to these diseases. It didn’t feel like it mattered physically, I only had poor digestion and my stomach was swollen.”(YA 08,51 years old, male patient).*

### Patients’ feeling when being diagnosed as hepatitis B

When patients were diagnosed with hepatitis B, they had two different kinds of feeling. Ten participants expressed their feelings very clearly in the interview. The three of them did not present any negative emotion towards the disease. As they explained, in the early stage of hepatitis B, they had no obvious symptoms and their lives were not affected, hence they could live and work as before. One participant described, “*at that time I didn’t know about the disease, I didn’t feel anything, and the doctor said it was nothing serious*”. *(YA 01,42 years old, female patient) “At that time, I did not know what hepatitis B was. I thought it was a minor disease, and I had no understanding about hepatitis B for many years.”(YA 41,55 years old, female patient) “I felt nothing. I do not feel any physical discomfort, so I did not pay any attention to it.”(YA 11,53 years old, female patient).*

Most of the patients talked about their anxiety, fear and depression due to the diseases. Patients experienced these feelings after they learned through the Internet and other means that hepatitis B is currently incurable. Besides, they were worried that the disease would be spread to their families especially their children. One patient said “*when I was found to have cirrhosis, I was getting scaled because one day it may become liver cancer. After all, I was still young, and my child had just started working.”(YA 09,44 years old, female patient)* However, for most of the patients, if their diseases were not very serious, they could accept the fact day by day. One patient recalled *“at that time, I was really depressed when I was found out to have hepatitis B because I searched online and found out that hepatitis B cannot be cured, and this made me scared. I was very afraid of it. But now I’m very optimistic and I’m willing to talk to someone about it.”(YA 36,50 years old, male patient).*

### Doctor’s advice to the patient

Doctors provided different advices to patients acorrding to stage of the disease. For patients who had no symptoms and their laboratory test indicated no liver damage, they were asked to take healthy food, have a good rest, and check their liver function per six months. Patients who needed treatment were requested to take medication on time and have regular medical check-ups to prevent from liver cancer. Interestingly, most patients were able to remember their doctor’s instructions about healthy diet or good rest, but they were not impressed by the doctor’s reminder of regular follow-up and liver function test. Dietary advice included less oil and salt, avoiding cold, hard, fried foods and drinking alcohol. As one interviewee said,*“at that time, the doctor told me that I was the carrier, it does not matter much, I need not to pay much attention. After the treatment started, the doctor ordered that I should pay attention to my diet. Do not eat cold or hard food, oily things, or things that are bad for my liver.”YA 01,(42 years old, female patient)* Another patient also got this advice *“In our daily life, we should not eat stimulating food, cold food, hard food or fat food.”(YA 39,54 years old, male patient)* Many patients could recall doctors’ suggestion on taking a good rest very clearly*. As one patient mentioned “the doctor told me we should live a regular life, take a good rest and do not engage in excessive physical activities.”* Another participant also reported *“the doctor asked me to take more rest, but should not drink nor stay up late, we should sleep well.”(YA 11,53 years old, female patient)* Very few patients could remember the doctors’ advice on regular check-ups. One patient pointed out this *“the doctor said I should take medical check-ups every six months.”(YA 42,50 years old, male patient).*

## Discussions

Although hepatitis B is a common disease in China and almost all individuals can afford to be screened, this study indicated that most cases were passively screened. Thus the low detection rate of HBV infection is not surprising [23, 24]. Prior to 2010, HBV testing was included in all kinds of entrance examinations and employee medical examinations. This has facilitated the detection of hepatitis B infection to some extent, but it has also increased the discrimination against hepatitis B in the society. The situation severely affected the study and work opportunities of HBV carriers without affecting the health of others. In 2010, the relevant state authorities explicitly eliminated the requirement for “HBV immunemarker testing” from medical examinations for school admission and employment. This policy aims at eliminating discrimination against hepatitis B and protecting the equal rights of hepatitis B patients. Consequently, hepatitis B will not be detected unless the patient has abnormal liver function, which in a public health sense, results in carriers not being detected in a timely manner. We suggest that residents should be informed of whether they have hepatitis B when their liver function is abnormal, and advocate that health workers promptly advise patients diagnosed with hepatitis B to seek medical care and treatment as soon as possible. At the same time, it is essential for health workers to provide health education to residents and encourage them to take the initiative to test for hepatitis B markers when their liver function is normal. Therefore, early detection, diagnosis, and treatment are very helpful in identifying patients in the healthy population and achieving secondary prevention [25, 26].

Our study found that more than half of hepatitis B patients have carriers in their homes. Some studies have also found that hepatitis B is significantly heritable in families [27, 28]. This suggests that our family members play an important role in hepatitis B screening. Therefore, in order to detect occult patients, we suggest a family-based diagnostic and treatment model. China is currently taking measures to increase public health investment and advocate extensive screening of residents (whole population strategy) to improve the detection rate of hepatitis B, and to achieve early detection, early diagnosis, and early treatment [29]. Compared with the population-wide strategy, we prefer the high-risk group strategy for hepatitis B prevention and management:1. Once there is a hepatitis B patient in the family, the patient himself and family members should pay attention to reduce the high-risk behaviors for transmitting hepatitis B, improve their consciousness, and strive to take corresponding measures as soon as possible to reduce the transmission of hepatitis B. 2. community workers and health workers should encourage residents to carry out hepatitis B screening, especially mobilizing high-risk groups for hepatitis B screening, which is helpful for improving the detection rate of hepatitis B [30, 31]. 3. Mutual supervision among extended family members facilitates adherence to hepatitis B follow-up and standardized treatment. The high-risk population strategy helps prevent hepatitis B, and early implementation of WHO’s goal of eliminating hepatitis B and reducing the incidence rate of hepatitis B in 2030 [32].

After the diagnosis of hepatitis B, it is also extremely important for doctors to remind patients to follow up for regular checkups. The research findings mainly include two aspects. On the one hand, hepatitis B patients were not impressed by their doctors’ advice about regular follow-up visits; what impressed them was their doctors’ advice about lifestyle and diet. On the other hand, many ignorant patients fail to adhere to regular follow-up for a long time, resulting in further deterioration of their disease [33]. Consequently, we recommend systematic training for doctors, including reminding patients of changes in their condition and emphasizing the importance of regular follow-up [34].

Another finding that should not be ignored is that the vast majority of patients are in a negative mood after diagnosis. The main reason is that the patients do not understand their disease and are not aware of hepatitis B. This requires clinicians to explain patiently to patients after diagnosis and to try to eliminate the impact of negative emotions on the patient’s follow-up [35], which will help the patient develop a healthy scientific perspective about hepatitis B. At present, there is a paucity of research on emotional changes in hepatitis B patients after diagnosis, and this needs to be further investigated. Our study has several limitations that must be acknowledged. First, the scale of the survey was small due to limited time, human and financial resources. Second, the participants in the survey were all patients from the hospital. Therefore, the conclusions drawn may not be generalizable to other regions of China, but they can provide some suggestions and recommendations for hepatitis B prevention.

## Conclusion

This study suggests that the patients’ lack of understanding about hepatitis B, the insufficient knowledge provided to hepatitis B patients at the time of diagnosis, and the failor of regular follow-up physical check-ups could lead to disease progression in the southwest of China.

Our findings suggest that there is a considerable gap between the early detection and timely treatment of hepatitis B and the current detection process of hepatitis B patients. There are a large number of problems and barriers in this setting, including inactive physical examinations and family carriers who are not actively prevented and treated, and insufficient impact of patients on follow-up and review, limiting the prevention and control of hepatitis B. Therefore, interventions are needed to improve the rate of detection of HBV infection. In addition, a focus should be placed on a family-based diagnostic model to focus on their condition as early as possible and to control further progression of the disease. Our findings also suggest that the training of health care workers should emphasize the importance of regular follow-up of hepatitis B and health education for the public.

## Data Availability

Authors are not able to share their raw data since participants disclosed very sensitive information. Participants were told that the transcripts will be read by interviewers only, and this prompted their trust. However, authors are willing to share codes generated from the data on request. All data generated or analysed during this study are included in this published article.
